# Changes in volume of stage I non-small-cell lung cancer during stereotactic body radiotherapy

**DOI:** 10.1186/1748-717X-9-8

**Published:** 2014-01-07

**Authors:** Kotoha Tatekawa, Hiromitsu Iwata, Takatsune Kawaguchi, Satoshi Ishikura, Fumiya Baba, Shinya Otsuka, Akifumi Miyakawa, Maho Iwana, Yuta Shibamoto

**Affiliations:** 1 Department of Radiology, Nagoya City University Hospital, 1 Kawasumi, Mizuho-cho, Mizuho-ku, Nagoya 467-8601, Japan; 2 Department of Radiation Oncology, Nagoya Proton Therapy Center, Nagoya West Medical Center, Nagoya, Japan; 3 Department of Radiation Oncology, Juntendo University, Urayasu, Tokyo, Japan; 4 Department of Radiation Oncology, Nagoya West Medical Center, Nagoya, Japan

## Abstract

**Background:**

The overall treatment time of stereotactic body radiotherapy (SBRT) for non-small-cell lung cancer is usually 3 to over 10 days. If it is longer than 7 days, tumor volume expansion during SBRT may jeopardize the target dose coverage. In this study, volume change of stage I NSCLC during SBRT was investigated.

**Methods:**

Fifty patients undergoing 4-fraction SBRT with a total dose of 48 Gy (*n* = 36) or 52 Gy (*n* = 14) were analyzed. CT was taken for registration at the first and third SBRT sessions with an interval of 7 days in all patients. Patient age was 29–87 years (median, 77), and 39 were men. Histology was adenocarcinoma in 28, squamous cell carcinoma in 17, and others in 5. According to the UICC 7th classification, T-stage was T1a in 9 patients, T1b in 27, and T2a in 14. Tumor volumes on the first and 8th days were determined on CT images taken during the exhalation phase, by importing the data into the Dr. View/LINAX image analysis system. After determining the optimal threshold for distinguishing tumor from pulmonary parenchyma, the region above -250 HU was automatically extracted and the tumor volumes were calculated.

**Results:**

The median tumor volume was 7.3 ml (range, 0.5-35.7) on day 1 and 7.5 ml (range, 0.5-35.7) on day 8. Volume increase of over 10% was observed in 16 cases (32%); increases by >10 to ≤20%, >20 to ≤30%, and >30% were observed in 9, 5, and 2 cases, respectively. The increase in the estimated tumor diameter was over 2 mm in 3 cases and 1–2 mm in 6. A decrease of 10% or more was seen in 3 cases. Among the 16 tumors showing a volume increase of over 10%, T-stage was T1a in 2 patients, T1b in 9, and T2a in 5. Histology was adenocarcinoma in 10 patients, squamous cell carcinoma in 5, and others in 1.

**Conclusions:**

Volume expansion >10% was observed in 32% of the tumors during the first week of SBRT, possibly due to edema or sustained tumor progression. When planning SBRT, this phenomenon should be taken into account.

## Background

Stereotactic body radiotherapy (SBRT) has become an important treatment option for stage I non-small-cell lung cancer (NSCLC) in recent years. Many reports have shown that SBRT is safe and effective for stage I NSCLC, since SBRT produces superior dose distribution within the target, while reducing the irradiated normal tissue volume compared with conventional radiotherapy [1-4]. However, the optimal dose fractionation schedule has not been established yet; a variety of schedules are being used at respective institutions, including 45–60 Gy in 3 or 4 fractions over 1–2 weeks and 55–65 Gy in 8 or more fractions over 2–3 weeks [5-9]. In Japan, 48 Gy delivered in 4 daily fractions has been the most frequently used schedule, as was used in the Japan Clinical Oncology Group (JCOG) study 0403 [10].

On the other hand, our group proposed a treatment protocol based on radiobiological background, employing different doses depending on tumor diameter and interfraction intervals of 3 days or longer [11-13]. The rationale for the strategy of twice weekly treatment was that the reoxygenation phenomenon of tumors could be better utilized by posing a longer interval between respective fractions [14,15]. With this strategy, however, the overall treatment time becomes longer, so changes in tumor size during the SBRT course may become a problem, since treatment plans are usually made only once before the start of treatment. In the present study, therefore, we evaluated tumor volume changes during SBRT for stage I NSCLC using a computer image analysis tool.

## Methods

### Study design and subjects

The study subjects were patients enrolled in a prospective SBRT study approved by the institutional review board of Nagoya City University Hospital (NCU-0401). Details and early clinical results of the study were reported previously [11-13]. Eligibility criteria of the study were as follows: (1) histologically confirmed primary NSCLC; (2) T1N0M0 or T2N0M0 disease according to the International Union Against Cancer (UICC) 1997 system by CT of the chest and upper abdomen, brain magnetic resonance imaging, and bone scintigraphy or 18-fluoro-deoxyglucose positron emission tomography; (3) greatest tumor dimension ≤ 5 cm; (4) World Health Organization performance status (PS) ≤ 2 or PS 3 when the cause was not a pulmonary disease; (5) no prior chest radiotherapy for the NSCLC to be treated by SBRT; (6) no active concurrent malignancy; and (7) written informed consent.

SBRT was delivered in 4 fractions, twice a week. According to the protocol, all patients treated at Nagoya City University Hospital underwent CT for registration at the first and third SBRT sessions. Fifty patients treated between July 2004 and August 2007 in whom the interval between the 1st and 3rd fractions was just 7 days were analyzed in this study (Table 1). Thirty-nine patients were male and 11 were female. Patient age ranged from 29 to 87 years (median, 77 years). Thirty-eight patients were medically inoperable and 12 refused surgery. Histology was adenocarcinoma in 28, squamous cell carcinoma in 17, and others in 5. Maximum tumor diameter ranged from 15 to 47 mm (median, 28 mm). According to the UICC 7th staging system, 9 patients had a T1a tumor, 27 had a T1b tumor, and 14 had a T2a tumor.

**Table 1:** Patient characteristics

	**All cases (n = 50)**	**Enlargement (+) (n = 16)**	**Enlargement (-) (n = 34)**	***P***
**Sex (male/female)**	39/11	14/2	25/9	0.31
**Age (years)**	77 (29–87)	76 (68–83)	77(29–87)	0.83
**Median (range)**				
**T-stage T1a/T1b/T2a**	8/28/14	2/9/5	6/19/9	0.87
**Tumor diameter (mm)**	28 (14–47)	27 (18–43)	28 (14–47)	0.84
**Median (range)**				
**Histology AD/SCC/others**	29/16/5	10/5/1	19/11/4	0.81

AD = adenocarcinoma, SCC = squamous cell carcinoma, others = non-small-cell carcinoma, not specified.

## SBRT methods

Our SBRT method has been described in detail previously [11-13]. Briefly, SBRT was performed using 3 coplanar and 4 non-coplanar static beams of 6-MV X rays from a linear accelerator (CLINAC 23EX, Varian Medical Systems, Palo Alto, California, USA). The prescribed total dose at isocenter was 48 Gy for T1a and T1b tumors and 52 Gy for T2a tumors, all given in 4 fractions. The total dose was 48 Gy in 36 patients and 52 Gy in 14. The Body Fix system (Medical Intelligence, Schwabmenchen, Germany) was used for patient immobilization.

### Evaluation of tumor volume

CT was taken just before the first and third treatments (days 1 and 8) under free-breathing conditions and breath holding during the exhalation and inhalation phases. For this study, CT images taken under breath holding during the exhalation phase were used because CT images at this phase were considered to be of the highest reproducibility in serial examinations. CT images were acquired using a multidetector-row scanner (MX-8000, Philips, Best, Netherlands) as described previously [16]. The scanning parameters were as follows: detector configuration, 2.5 × 4; slice thickness, 3.2 mm; increment, 2.5 mm; pitch, 0.875; rotation time, 0.75 sec; 120 kV; and 150 mAs/slice.

**Figure 1:**
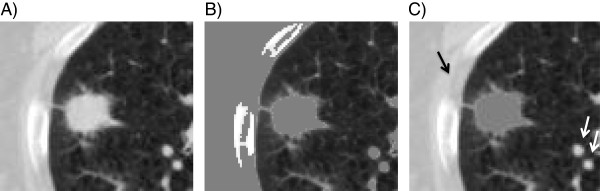
**Method to evaluate tumor volume using an image analyzing system, Dr. View/LINAX. (A)** First, the CT images were displayed at an optimal window level of -400 HU with a width of 1,400 HU. **(B)** The region above -250 HU was automatically extracted (gray areas). **(C)** The gray-painted structures outside the tumors such as vessels and chest walls (arrows) were manually excluded, and the tumor volume of the gray-painted regions was calculated.

All CT datasets were imported to the image analysis system, Dr. View/LINAX (AJS Inc., Tokyo, Japan), and analyzed with the window level setting appropriate for the lung (window width, 1,400 Hounsfield units, HU; window level, -400 HU). We carefully measured the CT number (HU) of lung tumors and pulmonary parenchyma, and determined the level of -250 HU as the optimal threshold that distinguishes between them. The region above -250 HU was automatically extracted and we then manually excluded the structures outside the tumors such as vessels and chest walls (Figure 1). Thereafter, tumor volumes were calculated using this system. Preliminarily, this procedure was repeated three times for 5 tumors selected randomly, and we confirmed that the tumor volumes were calculated within 3% variation. Tumor diameter was estimated assuming a spherical shape from the equation: volume = π/6 × (diameter)^3^.

### Statistical methods

Differences between pairs of groups were examined by *t*-test or Fisher’s exact test.

## Results

**Figure 2:**
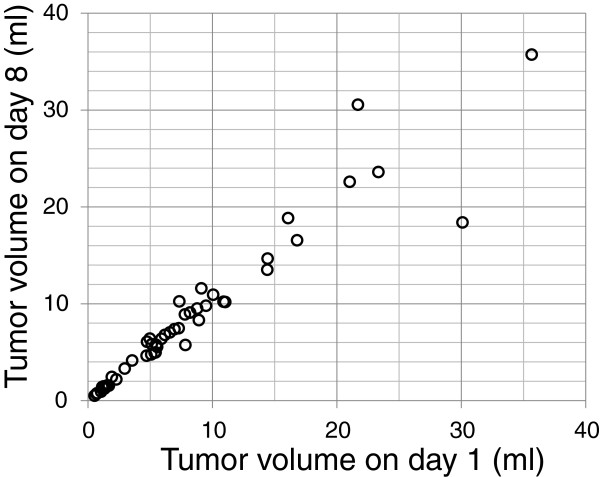
Tumor volumes on day 1 and day 8 in all 50 patients.

**Figure 3:**
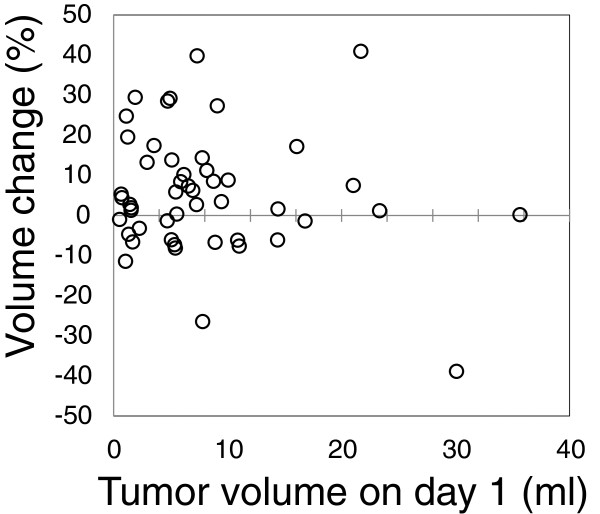
Relationship between tumor volume changes and tumor volume on day 1 in all 50 patients.

The median tumor volume was 7.3 ml (range, 0.5-35.7) on day 1 and 7.5 ml (range, 0.5-35.7) on day 8. Figure 2 shows the tumor volumes on days 1 and 8 in all 50 patients. Changes in the tumor volume and the tumor diameter estimated from the tumor volume are shown in Table 2. The relationship between tumor volume on day 1 and volume change is shown in Figure 3. A volume increase of over 10% was observed in 16 cases (32%); increases by >10 to ≤20%, >20 to ≤30%, and >30% were observed in 9, 5, and 2 cases, respectively. An increase of the estimated tumor diameter over 1 mm was observed in 9 patients (18%), among whom 3 (6%) showed an increase over 2 mm.

**Table 2:** Changes in tumor volume and diameter

**Change in volume (%)**	**n (%)**	**Change in diameter (mm)**	**n (%)**
≤ - 30	1 (2)	≤ - 3	1 (2)
> - 30, ≤ - 20	1 (2)	> - 3, ≤ - 2	1 (2)
> - 20, ≤ - 10	1 (2)	> - 2, ≤ - 1	1 (2)
> - 10, ≤0	13 (26)	> - 1, ≤0	13 (26)
>0, ≤10	17 (34)	>0, ≤1	25 (50)
>10, ≤20	9 (18)	>1, ≤2	6 (12)
>20, ≤30	5 (10)	>2, ≤3	2 (4)
>30	2 (4)	>3	1 (2)

A volume decrease of 10% or more was observed in 3 patients (6%); two had an adenocarcinoma and one had a squamous cell carcinoma. The tumor showing the greatest decrease of 38% was a squamous cell carcinoma. Three patients (6%) showed a decrease of 1 mm or more in the estimated diameter.

Characteristics of 16 patients showing more than 10% increase and 34 patients showing no increase are listed in Table 1. There were no differences in T-stage, tumor size, and distribution of histology between the two groups. For 29 adenocarcinomas, the volume change was 7.5 ± 14% (mean ± SD), while it was 0.8 ± 16% for 16 squamous cell carcinomas (*P* = 0.14).

## Discussion

In this study, we evaluated changes of tumor volume measured using an image-analyzing system, instead of the gross tumor volume (GTV) delineated manually in actual radiotherapy planning. The tumor volume measured on the basis of the CT number is smaller than the GTV in actual planning, since the system recognized only the solid parts on one phase CT image (expiratory phase) and did not contain spiculae and internal margins. As a result, we could objectively evaluate slight changes in tumor volume during the treatment. With this method, two issues exist regarding possible mismeasurement of the tumor volume. The first one is regarding delineation of the structures close to the tumor. Especially when the tumor is adjacent to the chest wall, it is sometimes difficult to separate the tumor and chest wall; in such cases, we drew the line along the anatomical location of the chest wall. We measured the same tumor three times and confirmed that the errors for the tumor volume estimation were within 3% variation. The second point is setting of the threshold to distinguish the lung parenchyma and tumor. Adequate CT numbers could differ among cases; the HU of regions like ground-glass opacity may be smaller than that for solid lesions. Nevertheless, we used -250 HU as the threshold in all cases because we apprehended that changing the threshold in respective cases would decrease the objectivity. We considered that using the same threshold would not be a major problem for the comparison of tumor volumes on the first and eighth days.

Decrease in tumor volume of 10% or more was observed in 3 (6%) patients. Previous studies indicated that squamous cell carcinomas regress faster than adenocarcinomas after Gamma Knife treatment or lung SBRT [17,18], but in the present study, 2 of the 3 tumors were adenocarcinomas while one was a squamous cell carcinoma. Rapid decrease of tumor volume during the course of SBRT may be due to apoptosis of the tumor cells [19]. In a study with murine tumors, adenocarcinomas tended to show more apoptosis after radiation than squamous cell carcinomas [20]. Decrease of tumor volume during the SBRT course may not pose a major problem, since this phenomenon only adds margins of a few millimeters in the latter half of the course.

On the other hand, an increase of tumor volume can adversely affect the dose distribution in PTV. According to other investigators, a GTV increase of >10 cm^3^ was observed during SBRT for NSCLC in two tumors in the first 2 weeks of treatment [21]. In addition, 25% increase of PTVs was observed on the repeat 4DCT scan [22]. In another study with 8 patients, slight increases and decreases in GTV appeared to occur in a few patients each, but overall GTV variations were not significant [23]. In all of these studies, the target volume was contoured manually. Although the fractionation schedule and prescribed dose are somewhat different, our study confirmed that lung cancers could show temporary enlargement in the first week during SBRT. We speculate that the main reason for this phenomenon may be edema due to high-dose irradiation, but sustained tumor progression can be another cause in rapidly growing tumors.

What is the influence on the dose coverage for the PTV? If sufficient margins are allowed at treatment planning, such changes may not pose a serious problem without making an adaptive treatment plan. In our SBRT studies, the CTVs were somewhat larger than the estimated tumor volumes, and we created the internal target volume (ITV) using fusion images of three CT phases. Furthermore, 5-mm margins were added to the ITV for the PTV. With our present treatment protocol, therefore, adaptive planning may not be necessary. Previous studies addressed the issue of appropriate margin settings and respiratory-gated irradiation using 4DCT [24-27]. These studies indicated that the use of 4DCT could reduce the PTV. In attempts at reducing the margin in the future, the phenomenon of volume expansion during SBRT course should be taken into account.

In summary, volume expansion over 10% was observed in 32% of the tumors during the first week of SBRT. In making SBRT plans, this phenomenon should be taken into account.

## Competing interests

The authors declare that they have no competing interests.

## Authors’ contributions

Each author participated sufficiently in the work to take public responsibility for appropriate portions of the content. HI and SI designed the study. TK and KT measured the tumor volume. KT wrote the manuscript; all other authors helped. All authors read and approved the final manuscript.
